# Influence of Biometric and Corneal Tomographic Parameters on Normative Corneal Aberrations Measured by Root Mean Square

**DOI:** 10.3390/jcm13237125

**Published:** 2024-11-25

**Authors:** Ignacio Almorín-Fernández-Vigo, Silvia Pagán Carrasco, Inés Sánchez-Guillén, José Ignacio Fernández-Vigo, Ana Macarro-Merino, Bachar Kudsieh, José Ángel Fernández-Vigo

**Affiliations:** 1 Centro Internacional de Oftalmología Avanzada, 06011 Badajoz, Spain; 2 Department of Ophthalmology, Hospital Universitario Rafael Mendez, 30817 Lorca, Spain; 3 Department of Ophthalmology, Hospital Perpetuo Socorro, 06010 Badajoz, Spain; 4 Centro Internacional de Oftalmología Avanzada, 28010 Madrid, Spain; 5 Department of Ophthalmology, Hospital Clínico San Carlos, 28040 Madrid, Spain; 6 Department of Ophthalmology, Hospital Puerta de Hierro, 28220 Madrid, Spain; 7 School of Medicine, Universidad de Extremadura, 06006 Badajoz, Spain

**Keywords:** corneal aberrations, corneal tomography, high-order aberrations, low-order aberrations, keratoconus, Scheimpflug tomography

## Abstract

**Background/Objectives**: To determine the impact of corneal and biometry parameters on the normative root mean square (RMS) values of corneal aberrations measured at a 6 mm diameter. **Methods**: The RMS values for corneal aberrations (anterior, posterior, and total) were measured along with corneal parameters using Scheimpflug tomography on 770 normal subjects. The biometric parameters were measured with an optical biometer. A multiple linear regression model was used to assess the effect of these parameters on the RMS values for corneal aberrations. **Results**: The mean RMS values for low-order (LOAs) and high-order 6 mm aberrations (HOAs) were 1.883 ± 0.797 µm and 0.484 ± 0.173 µm, respectively, and for the anterior and posterior cornea, the values were 0.775 ± 0.166 µm and 0.189 ± 0.036 µm, respectively. For the anterior cornea, the main predictors of the RMS LOAs (R^2^ = 69.8%) were anterior corneal astigmatism (ACA) and anterior corneal elevation (Ele F) on the apex (both *p* < 0.0001) and for the RMS HOAs (R^2^ = 33.3%) the main predictors were age, ACA, and Ele F on the thinnest point (all *p* < 0.0001). For the posterior cornea, considering only the posterior corneal variables, the main predictors of the RMS LOAs (R^2^ = 63.4%) were posterior corneal astigmatism and posterior corneal elevation (Ele B) on the thinnest point and apex (all *p* < 0.0001) and for the RMS HOAs (R^2^ = 46%) the main predictors were the mean posterior keratometry and Ele B on the thinnest point and apex (all *p* < 0.0001). **Conclusions**: Normative data of RMS values for corneal aberrations measured over 6 mm are influenced by age and several corneal parameters, which should be considered when evaluating the diagnostic ability of the RMS values.

## 1. Introduction

Corneal refractive surgery with laser vision correction (LVC) has recently gained increasing interest as an effective fast-recovery procedure, mainly because of its safety [1]. Currently, most research efforts are intended to achieve better laser performance and more precise exclusion criteria to detect subclinical keratoconus and diminish the risk of developing progressive corneal ectasia [2].

Several tomographic corneal parameters have been described to increase during the development of corneal ectasia, including the pachymetry progression index, corneal elevations, and curvature asymmetry [3]. Derived from elevation data, corneal monochromatic aberrations have also been used as screening parameters in diagnosing clinical and subclinical keratoconus (KC) [4] and even as a KC grading scale [5].

The corneal wavefront aberration function can be calculated for the cornea’s anterior and posterior surfaces or both surfaces (total cornea) from corneal tomography data. Then, the wavefront analysis can be mathematically decomposed and represented with Zernike polynomials to weigh the contribution of each polynomial function to the total amount of optical aberration. When evaluating optic aberrations with Zernike decomposition, they can be divided into LOAs (up to 2nd order) or HOAs (3rd and above orders) [6]. Additionally, other metrics can be derived to describe the image quality, such as the root mean square (RMS) error, which quantifies the optical quality of the cornea [6]. These corneal aberration descriptors should refer to the corneal surface measured (anterior, posterior, or total) and their diameter [7,8], as those factors yield different measurements. In addition, several studies found other parameters to be responsible for corneal aberration measurement variability, with age being the primary predictor [9,10,11,12]. However, for race, the results were controversial [13,14,15]. Nevertheless, no other parameters have been simultaneously studied to detect their combined influence on corneal aberrations. As this could lead to the setting of incoherent threshold values when screening for KC, we designed a study to describe the normative RMS values of anterior, posterior, and total corneal aberration measurements at 6 mm diameter in a large Caucasian population and to assess the impact of corneal and biometric parameters on them.

## 2. Materials and Methods

This study was observational, analytical, cross-sectional, and retrospective.

**Figure 1:**
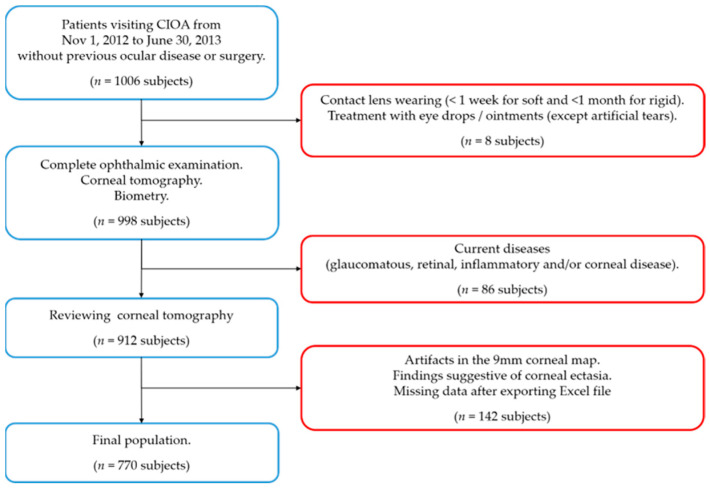
Flow chart for the inclusion (blue squares) and exclusion criteria (red squares). n = number of subjects.

Participants attending the Centro Internacional de Oftalmología Avanzada (CIOA, Madrid, Spain) over eight months (between 1 November 2012 and 30 June 2013) were consecutively evaluated by a routine ophthalmology exam (Figure 1), as previously described [16]. The tenets of the Declaration of Helsinki were followed. The study protocol and procedures received Review Board approval from the CIOA. All participants must have signed their written informed consent.

Patients with no history of ocular disease and/or surgery and who were collaborative enough to be properly evaluated with complementary tests were included. The exclusion criteria were as follows: contact lens wearing (<1 week for soft and <1 month for rigid gas permeable), current treatment with any eye drops (except for artificial tears), and a glaucomatous, retinal, inflammatory, and/or corneal pathology (congenital or acquired, including leukomas) detected during the study examination. Subjects were also excluded if corneal ectasia was suspected in corneal tomography according to the following indications: an asymmetric bow-tie pattern, an abnormal steepening or skewed radial axis [2], or the combination of a rate of progression of pachymetry of more than 1.2 with the thinnest point less than 450 µm accompanied by a posterior elevation greater than 13.5 µm [17].

The following examinations were performed on all subjects: best-corrected distance visual acuity with subjective and cycloplegic refraction, biomicroscopy of the anterior segment, corneal tomography with the Pentacam (Oculus Optikgeräte GmbH, Wetzlar, Germany), biometry with the IOLMaster 500 (Carl Zeiss Meditec Inc., Dublin, CA, USA), pneumatic non-contact tonometry with the Canon TX 10^®^ (Canon Inc., Tokyo, Japan), and a fundus exam following pupil dilation. Age and sex were also recorded.

Axial length (AXL) and horizontal corneal diameter as white-to-white distances (WTW) were measured in mm by partial coherence interferometry using the IOL Master 500 (Carl Zeiss Meditec Inc., Dublin, CA, USA). Both were mean values of repeated measurements (ten valid measurements from up to twenty attempts for AXL and three measurements for WTW).

Corneal tomography was performed with a Pentacam single rotation Scheimpflug camera (Oculus Optikgeräte GmbH, Wetzlar, Germany): the capture rate was 25 B-scans in 2 s, and the mode was automatic release. Only tomographs with a sufficient quality score (labeled by the software as “OK”) and at least 9 mm in diameter without extrapolated data were accepted, so only real valid measurements were included. The variables extracted from the Pentacam were as follows: anterior chamber depth from endothelium in mm (ACD), mean anterior and posterior keratometry (KmF and KmB, respectively), astigmatism (ACA and PCA, respectively) in a 3 mm ring diameter in D, anterior and posterior corneal asphericity (QF and QB, respectively) in a 6 mm zone diameter, pachymetry at the thinnest point of the cornea (Thinnest) in µm, and anterior and posterior corneal elevation data measured in µm at the apex (Ele F Apex and Ele B Apex, respectively) and the thinnest point of the cornea (Ele F Thin and Ele B Thin, respectively) in relation to a float best-fit-sphere with a fixed diameter of 8 mm, as described in the literature [18]. Additionally, anterior, posterior, and total corneal aberrations were measured at a 6.0 mm diameter from the Zernike maps and evaluated as the RMS values for low-order aberrations (RMS LOAs), high-order aberrations (RMS HOAs), and total aberrations (RMS total).

The software used in the statistical analysis was the Statistical Package for Social Sciences for Windows (SPSS v25, IBM Corp. Armonk, NY, USA).

The number and percentage of cases in each category were presented for the qualitative variables, and the mean values ± standard deviation, range, and percentiles were shown for the quantitative variables. The normal distribution of data was checked by the Kolmogorov–Smirnov test.

Two multiple linear regression (MLR) models were created to evaluate the effects of the independent variables on the RMS total, RMS HOAs, and RMS LOAs from the anterior, posterior, or total cornea as dependent variables. Significance was set at *p* ≤ 0.05. Both models were formed with sex, age, sphere, refractive astigmatism (RA), WTW, AXL, Thinnest, and ACD as the constant independent variables. Additionally, the corneal parameters from the anterior (KmF, ACA, QF, Ele F Apex, and Ele F Thin) and posterior cornea (KmB, PCA, QB, Ele B Apex, and Ele B Thin) were used as independent variables, reliant on the model. Then, the model’s fit was evaluated through the determination coefficient (R2).

A first MLR (model 1) was formed with the constant variables and anterior corneal parameters. Model 1 was applied to all the RMS values for corneal aberrations from the anterior, posterior, and total cornea.

A second MLR (model 2) was formed with the constant variables and posterior corneal parameters. Model 2 was applied to all the RMS values for corneal aberrations only from the posterior cornea.

## 3. Results

After examination, 770 subjects remained as the total sample. Only the right eye of each subject was included. The mean values and range are provided for several variables with non-normal distributions for comparability with other articles. The mean age was 50.5 ± 15 years (range of 17 to 93 years); in the sample, 63.2% were women. The mean values for the sphere and RA were −0.41 ± 3.46 D (from −14.5 to + 10.25 D) and 0.86 ± 0.9 D (from 0 to 6 D), respectively, with 302 subjects showing as myopic, 312 as hyperopic, and 158 as emmetropic. The mean AXL and WTW values were 23.91 ± 1.56 mm (from 20.24 to 32.59 mm) and 12.09 ± 0.6 mm (from 10.9 to 13.4 mm), respectively.

The majority of the variables were not normally distributed (*p* ≤ 0.045), except for ACD (*p* = 0.056), Thinnest (*p* = 0.062), KmF (*p* = 0.2), and KmB (*p* = 0.2). The mean ACD value was 2.79 ± 0.43 mm (from 1.52 to 4.09 mm), the mean Thinnest value was 546.54 ± 33.02 µm (from 451 to 641 µm), and the mean KmF and KmB values were 43.9 ± 1.43 D (from 38.85 to 47.8 D) and −6.3 ± 0.24 D (from −7.0 to −5.3 D), respectively. Supplemental Table S1 (Online Resource) shows the median and interquartile range from all the non-normally distributed variables.

The mean 6 mm RMS total, RMS LOAs, and RMS HOAs were 1.952 ± 0.798 µm, 1.883 ± 0.797 µm, and 0.484 ± 0.173 µm, respectively, for the anterior cornea. The mean 6 mm RMS total, RMS LOAs, and RMS HOAs were 0.799 ± 0.166 µm, 0.775 ± 0.166 µm, and 0.189 ± 0.036 µm, respectively, for the posterior cornea. The mean 6 mm RMS total, RMS LOAs, and RMS HOAs were 1.673 ± 0.759 µm, 1.599 ± 0.756 µm, and 0.464 ± 0.180 µm, respectively, for the total cornea.

Since the data of the multivariate regression models between the RMS total and RMS LOAs were nearly identical, only the RMS LOAs data are presented for anterior, posterior, or total cornea. Similarly, since the data for the RMS total and RMS LOAs between the anterior and total cornea were nearly identical, only the data for the anterior cornea are presented.

Therefore, the data of model 1 for RMS LOAs are provided for the anterior and posterior cornea (Table 1 and Table 2, respectively), and RMS HOAs for the anterior and posterior cornea (Table 3 and Table 4, respectively).

Finally, the data of model 2 for the RMS LOAs (Table 5) and RMS HOAs (Table 6) are provided for the posterior cornea.

The tables not included in the manuscript, because of their similarity with those already presented, are available as supplemental materials in the online resources (Supplemental Tables S2–S4 for model 1 for the RMS total, RMS LOAs, and RMS HOAs of the total cornea, respectively. Supplemental Tables S5 and S6 for model 1 for the RMS total of the anterior and posterior cornea, respectively. Supplemental Table S7 for model 2 for the RMS total of the posterior cornea).

## 4. Discussion

Published data on RMS corneal aberrations can show wide variations between studies, which can be explained by methodological differences (such as different diameter measurements, or devices used for measurement) or population characteristics. Our sample, encompassing a wide age range and representative refractive error distribution of a Caucasian population, aims to minimize these population-derived variabilities.

Our multivariable regression models for the total RMS and low-order aberrations (RMS LOAs) yielded nearly identical results across all models, indicating that while high-order aberrations (RMS HOAs) were described as partially compensating for the LOA effects, the LOA remains the primary contributor to the total aberrations. Similarly, our model 1 (which uses anterior corneal parameters) for the RMS total, LOAs, and HOAs between the anterior and total cornea were, again, nearly identical, emphasizing that most total corneal aberrations originate from the anterior cornea [6] under non-pathological conditions. This generalization, however, may not apply to pathological or surgically altered corneas, as some situations can lead to differences in only one surface of the cornea (posterior corneal ectasia, LVC, and pterygium or corneal scars, etc.). Therefore, we will discuss the RMS total and LOAs altogether and the HOAs separately.

In our regression model 1, for the anterior cornea, the RMS LOA increase was mainly due to an increasing ACA and age and a diminishing anterior elevation in the apex, RA, and QF. This was also found for the total cornea and the RMS total. It should be mentioned that ACA and RA have opposite effects in our model, as RA is expressed as negative. This model predicts nearly 70% of the variability of the RMS LOAs and the total. As long as the RMS LOA is formed by up to second-order aberrations, in which astigmatism (Z2-1 and Z21) is one of the main components, ACA and RA play crucial roles, consistent with prior findings [19,20]. Only one article found a mild correlation between PCA and the RMS total and LOAs of the total cornea [21], which could explain why RA remains a moderate predictor even in the presence of ACA in our model 1 for the total cornea. This relationship reflects the importance of minimizing the astigmatism magnitude in any refractive procedure as a priority to reduce LOAs and improve visual quality, knowing that age will progressively decrease optical quality.

For the posterior cornea, model 1 was applied to predict measurements of the posterior corneal RMS total and LOAs with anterior corneal parameters, as most topographers only use those, achieving a modest predicting capability of over 30%. In this case, ACA and KmF have a direct effect and anterior elevation on the apex and age (in contrast to the anterior cornea) have an indirect effect. While clinically less relevant in normal corneas—where anterior aberrations dominate—this relationship may become an important finding for corneas with a greater contribution of the posterior surface (such as after LVC or ectasia) and warrants further investigation in corneas with abnormal anterior-posterior relations. In contrast, model 2 (using posterior corneal parameters) demonstrated a greater predictive power for the posterior cornea RMS total and LOAs (R^2^ > 60%). Key predictors included posterior elevation on apex and KmB (indirect effects), and posterior elevation on Thinnest, PCA, and QB (direct effects). These results highlight PCA as a significant contributor to posterior corneal aberrations, akin to ACA for the anterior cornea.

As we mentioned before, the RMS HOAs were predicted differently depending on whether the anterior (model 1) or posterior (model 2) models were used:

We found that in model 1, the RMS HOAs of the anterior and total cornea increased mainly with increasing age. Despite a brief report [22], it has been widely described that the RMS HOAs of the anterior [8,23,24,25,26] and total cornea [26,27] increase with age, although some authors specifically found an initial decrease from 40 to 60 years [23] with a marked following increase [27]. This probably explains the higher central tendency measurements of RMS HOAs of the anterior cornea found in some studies (mean values of 0.65 ± 0.38 [23] and a median of 0.552 [24]) with older populations and a slightly lower value of 0.453 ± 0.194 [27] with younger populations than those in our study. However, other parameters seem to be involved. In our study, along with age, ACA, QF, and KmF presented a direct effect and the anterior elevation on Thinnest and WTW presented an indirect effect in model 1, with a prediction capability of 33 to 39.5%. Other studies, despite differences in the measurement area and corneal topographer used, also found the same tendency for ACA [20,23,25]. Regarding KmF, with MLR analysis, one study found the same direct effect [25], but the other found an indirect effect [23]. This controversy could be explained by the fact that the latter did not include ACA in their MLR model. However, they also detected central corneal thickness (CTT) to be statistically significant but with less effect magnitude. Using other corneal and biometry variables may lead to CCT losing significance, as we did not find any pachymetric parameter to be involved in any of the RMS groups studied.

For the RMS HOAs of the posterior cornea, model 1 only achieved a mild prediction capability of nearly 20%, limiting its clinical applications. Instead, model 2 presented a 46% prediction capability, with the posterior elevations on the apex and KmB as predictors with indirect effects and the posterior elevation on the Thinnest as a predictor with a direct effect. It seems that posterior corneal elevations influence both LOA and HOA posterior corneal aberrations, which may reflect their importance as an irregularity parameter for the posterior cornea.

Corneal aberration data have been used in several studies for different purposes such as measuring corneal image quality [20], finding candidates for IOL-correcting presbyopia [28], or detecting early corneal ectasia cases [4,29,30]. However, as corneal aberration measurements showed variations according to the measurement device [31,32], ethnic group [33], preterm birth [34,35], and age [9,27], local normative data are needed to minimize the variability of the results. In the same way, studying the physiological relations between corneal aberrations and other corneal and ocular biometric parameters may help to better determine the diagnostic performance of some indices when suspecting corneal ectasia. These relations, for example, were previously studied and described for the anterior and posterior corneal elevation measurements [16,18]. Regarding one of these parameters, the subjective refraction, our results showed that the sphere was a poor predictor in model 1 only for the RMS HOAs of the anterior cornea. Similar findings were seen in the literature only for hyperopia between +1 and +4D [36]. Other authors did not find any correlation between spherical refraction and the RMS HOAs of the anterior [19,37] or posterior cornea [36] when evaluating both parameters alone. On the other hand, our study showed the importance of RA as one of the main predictors for the RMS total and LOAs even in the presence of ACA and PCA. In contrast, RA did not appear to have any effect on RMS HOAs. Contrary to our results, two authors found RMS HOAs to increase in higher astigmatic subjects [19,38]. As they did not use a multivariable model, only the effect of RA was evaluated, which may be confused by ACA, as it was a better predictor in our model.

Several studies have evaluated the performance of some aberration parameters in detecting early KC by comparing their values with a control normal population and obtaining cut-off values for the best area under the curve (AUC) in the receiver operating characteristic (ROC) curves. Nevertheless, the selection of the normal population has critical importance as their values in some parameters could vary the cut-off values if they are not correctly matched and compensated. Reddy et al. compared 23 early KC and 49 non-KC patients and found that the RMS HOAs of the total cornea were nearly twofold higher in early KC than in non-KC (2.48 ± 1.58 vs. 1.25 ± 0.61, respectively), which yielded a cut-off value of 1.15 for an AUC of 0.82 with 96% sensitivity and 58% specificity [4]. Both groups had no differences between age and sphere (*p* > 0.05), but the RA was three times higher in early KC than in non-KC (2 ± 1.8 D vs. 0.62 ± 0.68 D) [4]. As our regression model in normal patients for RMS HOAs for the total cornea (Supplemental Table S4) showed, ACA (which is correlated with RA) was the third main predictor, with a positive effect (the higher the ACA, the higher the RMS HOAs). This means that differences between groups in the RMS HOAs of the total cornea are, in a certain percentage, derived from the astigmatism differences, leading to an overestimation of the effect. As such, a more precise lower cut-off value should be expected if groups were also matched by RA. Other authors have also studied, in a similar article, the performance of RMS HOAs of the anterior cornea and found a paradoxically higher value for non-KC than for early KC (1.61 ± 0.63 vs. 1.48 ± 0.5, respectively) [29]. Although differences between groups were not statistically studied, the non-KC group had an older mean age (38 ± 9 vs. 30 ± 9, respectively) and a greater RA (−1.11 ± 0.92 vs. −0.53 ± 0.55) [29]. Again, based on our regression model in normal patients for RMS HOAs of the anterior cornea (Table 3), age and ACA were the first and third main predictors with a positive effect. Matching by age and RA should lead to a lower value for the RMS HOAs of the anterior cornea, which would be in accordance with previous findings [5]. Reviewing the literature, several other articles that evaluated the performance of the RMS HOAs of the anterior, posterior, or total cornea continue matching only age, but not RA or ACA [39,40,41,42,43,44], and some did not even match age in all groups [30,42].

Lastly, based on our findings, we propose some clinical recommendations:

Future studies regarding ectasia screening should fit the control group (normal corneas) to the main predictors identified in our study to match those parameters with the case group (early KC), as in the study by Heidari, which matched age and RA [45], leading to values with more precise cut-off values.

This study also highlights the strong association between age and RMS values, particularly in the anterior cornea, where age-related increases in LOAs and HOAs can lead to a decline in visual quality, which may be especially important after refractive surgery. For older patients, personalized approaches like wavefront-guided LASIK may be necessary to address these aberrations effectively. In contrast, younger patients typically exhibit fewer aberrations, allowing for more predictable refractive outcomes and greater flexibility in surgical decisions. Age should thus be a key factor in preoperative evaluations, guiding treatment choices to ensure optimal visual quality across different age groups.

Our study has several limitations. First, the accommodative status during the Pentacam examination was not assessed. This could lead to underestimating the HOA values if the patient was accommodating, although the effect was described to be low [46]. Second, as it was cross-sectional, the effects of age can only be inferred, highlighting the need for longitudinal studies to directly evaluate this impact. Additionally, there is a possibility that subclinical ectasia cases were unintentionally included, as definitive topographic criteria to rule out ectasia are not yet established. This is particularly challenging in younger individuals, who may be more prone to undetectable ectasia. Last, as only the right eye was studied, the findings can only be extrapolated to the contralateral eye.

## 5. Conclusions

In conclusion, our study provides normative RMS total, LOA, and HOA data of the anterior, posterior, and total cornea for a Caucasian population. Several corneal parameters and age affect the values of the corneal aberrations measured in terms of RMS, especially RMS total and LOAs, in a normal population. This relation should be taken into account when screening for early KC suspects using aberration data.

## Figures and Tables

**Table 1:** Multivariant regression model 1 for the RMS LOAs of the anterior cornea.

Sig.	t	Standardized Coefficients	Unstandardized Coefficients	Dependent Variables Predicting RMS LOAs (Anterior Cornea)
		Beta	B	
0.933	−0.084		−0.082	(Constant)
0.000 *	23.838	0.660	0.661	ACA
0.000 *	7.454	0.171	0.009	Age
0.000 *	−10.746	−0.387	−0.270	Ele F Apex
0.000 *	−6.745	−0.178	−0.158	RA
0.003 *	−3.014	−0.070	−0.137	WTW
0.000 *	−4.798	−0.163	−1.021	QF
0.000 *	4.242	0.106	0.058	KmF
R^2^ (%) = 69.8% Model F = 247.796				

* Statistically significant (*p* < 0.05).

**Table 2:** Multivariant regression model 1 for the RMS LOAs of the posterior cornea.

Sig.	t	Standardized Coefficients	Unstandardized Coefficients	Dependent Variables Predicting RMS LOAs (Anterior Cornea)
		Beta	B	
0.000 *	−3.540		−0.601	(Constant)
0.000 *	9.731	0.311	0.065	ACA
0.000 *	9.307	0.306	0.036	KmF
0.000 *	−9.360	−0.315	−0.004	Age
0.000 *	−3.922	−0.124	−0.043	Sex
0.000 *	−4.677	−0.218	−0.032	Ele F Apex
0.000 *	3.625	0.162	0.016	Ele F Thin
R^2^ (%) = 31.3% Model F = 57.772				

* Statistically significant (*p* < 0.05).

**Table 3:** Multivariant regression model 1 for the RMS HOAs of the anterior cornea.

Sig.	t	Standardized Coefficients	Unstandardized Coefficients	Dependent Variables Predicting RMS LOAs (Anterior Cornea)
		Beta	B	
0.122	1.547		0.487	(Constant)
0.000 *	8.266	0.284	0.003	Age
0.000 *	3.968	0.159	0.212	QF
0.000 *	5.830	0.224	0.048	ACA
0.000 *	−6.288	−0.258	−0.025	Ele F Thin
0.000 *	−3.976	−0.140	−0.058	WTW
0.004 *	2.894	0.103	0.012	KmF
0.025 *	2.246	0.074	0.004	Sphere
0.033 *	2.139	0.067	0.024	Sex
R^2^ (%) = 33.3% Model F = 47.649				

* Statistically significant (*p* < 0.05).

**Table 4:** Multivariant regression model 1 for the RMS HOAs of the posterior cornea.

Sig.	t	Standardized Coefficients	Unstandardized Coefficients	Dependent Variables Predicting RMS LOAs (Anterior Cornea)
		Beta	B	
0.000 *	−7.098		−0.264	(Constant)
0.000 *	12.915	0.441	0.011	KmF
0.000 *	−7.136	−0.256	−0.001	Age
0.000 *	−4.071	−0.148	−0.005	Ele F Apex
R^2^ (%) = 19.9% Model F = 62.794				

* Statistically significant (*p* < 0.05).

**Table 5:** Multivariant regression model 2 for the RMS LOAs of the posterior cornea.

Sig.	t	Standardized Coefficients	Unstandardized Coefficients	Dependent Variables Predicting RMS LOAs (Anterior Cornea)
		Beta	B	
0.000 *	−4.074		−0.462	(Constant)
0.000 *	16.572	0.431	0.432	PCA
0.000 *	7.792	0.358	0.279	QB
0.000 *	−11.223	−0.290	−0.197	KmB
0.000 *	11.200	0.448	0.013	Ele B Thin
0.000 *	−9.487	−0.518	−0.026	Ele B Apex
0.000 *	−5.061	−0.144	−0.002	Age
0.004 *	−2.907	−0.068	−0.024	Sex
0.010 *	−2.578	−0.062	−0.011	RA
R^2^ (%) = 63.4% Model F = 144.297				

* Statistically significant (*p* < 0.05).

**Table 6:** Multivariant regression model 2 for the RMS HOAs of the posterior cornea.

Sig.	t	Standardized Coefficients	Unstandardized Coefficients	Dependent Variables Predicting RMS LOAs (Anterior Cornea)
		Beta	B	
0.000 *	−10.596		−0.295	(Constant)
0.000 *	−16.170	−1.064	−0.012	Ele B Apex
0.000 *	−18.005	−0.536	−0.079	KmB
0.000 *	12.079	0.556	0.004	Ele B Thin
0.005 *	−2.810	−0.093	0.000	Age
0.041 *	−2.050	−0.113	−0.019	QB
R^2^ (%) = 46.0% Model F = 128.154				

* Statistically significant (*p* < 0.05).

## Data Availability

The data presented in this study are available on request from the corresponding author due to privacy concerns.

## Supplementary Materials

The following supporting information can be downloaded at: https://www.mdpi.com/article/10.3390/jcm13237125/s1, Table S1: Median and interquartile range (IQR) of the variables not normally distributed; Table S2: Multivariant linear regression Model 1 for the RMS total of the total cornea; Table S3: Multivariant regression Model 1 for the RMS LOAs of the total cornea; Table S4: Multivariant regression Model 1 for the RMS HOAs of the total cornea; Table S5: Multivariant regression Model 1 for the RMS total of the anterior cornea; Table S6: Multivariant regression Model 1 for the RMS total of the posterior cornea; Table S7: Multivariant regression Model 2 for the RMS total of the posterior cornea. Table S8. Multivariant regression Model 3 for the RMS total of the total cornea; Table S9. Multivariant regression Model 3 for the RMS LOA of the total cornea.
