# Supplementary material for: Influence of Biometric and Corneal Tomographic Parameters on Normative Corneal Aberrations Measured by Root Mean Square
Source: J Clin Med. 2024 Nov 25;13(23):7125. doi: 10.3390/jcm13237125 (PMC11642347; doi:10.3390/jcm13237125)
Supplement: Supplementary file 1 [file jcm-13-07125-s001.zip › jcm-3260680-supplementary.pdf]

Table S1.- Median and interquartile range (IQR) of the variables not normally distributed.

	Median	IQR
Age (years)	51.00	23.75
Sphere (D)	0.25	4.00
RA (D)	-0.75	1.00
WTW (mm)	12.1	0.60
AXL (mm)	23.70	1.88
ACA (D)	0.90	0.90
PCA (D)	0.32	0.20
QF	-0.23	0.17
QB	-0.24	0.28
Thinnest (µm)	546	42.00
Ele F Apex (µm)	2	2.00
Ele F Thin (µm)	2	2.00
Ele B Apex (µm)	3	5.00
Ele B Thin (µm)	7	7.00
RMS (Anterior cornea):	1.804	0.90
RMS HOA (Anterior cornea):	0.453	0.17
RMS LOA (Anterior cornea):	1.742	0.89
RMS (Posterior cornea):	0.786	0.21
RMS HOA (Posterior cornea):	0.185	0.04
RMS LOA (Posterior cornea):	0.763	0.21
RMS (Total cornea):	1.527	0.88
RMS HOA (Total cornea):	0.429	0.19
RMS LOA (Total cornea):	1.458	0.89

Table S2.- Multivariant linear regression Model 1 for the RMS total of the total cornea.

Dependent variables predicting RMS total (Total cornea)	Unstandardized coefficients	Standardized coefficients	t	Sig.
	B	Beta		
(Constante)	0.956		0.923	0.356
ACA	0.564	0.591	18.458	0.000*
Age	0.015	0.279	11.005	0.000*
Ele F Apex	-0.199	-0.300	-6.780	0.000*
RA	-0.179	-0.213	-7.222	0.000*
WTW	-0.180	-0.097	-3.747	0.000*
Ele F Thin	-0.063	-0.143	-4.280	0.000*
QF	-1.049	-0.176	-4.623	0.000*
KmF	0.038	0.070	2.618	0.009*

R^2 (%)= 63.0%   Model F = 159.638

* Statistically significant ($p<0.05$)

Table S3.- Multivariant regression Model 1 for the RMS LOA of the total cornea.

Dependent variables predicting RMS LOA (Total cornea)	Unstandardized coefficients	Standardized coefficients	t	Sig.
	B	Beta		
(Constante)	0.764		0.746	0.456
ACA	0.568	0.598	18.814	0.000*
Age	0.014	0.268	10.643	0.000*
Ele F Apex	-0.206	-0.311	-7.072	0.000*
RA	-0.182	-0.217	-7.419	0.000*
WTW	-0.169	-0.092	-3.562	0.000*
QF	-1.144	-0.193	-5.096	0.000*
Ele F Thin	-0.058	-0.133	-3.998	0.000*
KmF	0.038	0.071	2.638	0.009*

R^2 (%)= 63.5%   Model F = 163.134

* Statistically significant ($p<0.05$)

Table S4.- Multivariant regression Model 1 for the RMS HOA of the total cornea.

Dependent variables predicting RMS HOA (Total cornea)	Unstandardized coefficients	Standardized coefficients	t	Sig.
	B	Beta		
(Constante)	1.253		7.513	0.000*
Age	0.005	0.377	11.898	0.000*
Ele F Thin	-0.030	-0.291	-7.484	0.000*
ACA	0.051	0.231	7.670	0.000*
WTW	-0.070	-0.162	-5.178	0.000*
QF	0.172	0.124	3.262	0.001*
Sex	0.029	0.077	2.629	0.009*
AXL	-0.007	-0.064	-2.003	0.046*

R^2 (%)= 39.5% Model F = 70.605

* Statistically significant ($p<0.05$)

Table S5.- Multivariant regression Model 1 for the RMS total of the anterior cornea.

Dependent variables predicting RMS total (Anterior cornea)	Unstandardized coefficients	Standardized coefficients	t	Sig.
	B	Beta		
(Constante)	0.089		0.090	0.928
ACA	0.683	0.652	23.286	0.000*
Age	0.010	0.179	7.713	0.000*
Ele F Apex	-0.268	-0.384	-10.531	0.000*
RA	-0.156	-0.176	-6.590	0.000*
WTW	-0.147	-0.075	-3.196	0.001*
QF	-0.924	-0.148	-4.287	0.000*
KmF	0.058	0.107	4.222	0.000*

R^2 (%)= 69.1% Model F = 239.402

* Statistically significant ($p<0.05$)

Table S6.- Multivariant regression Model 1 for the RMS total of the posterior cornea.

Dependent variables predicting RMS total (Posterior cornea)	Unstandardized coefficients	Standardized coefficients	t	Sig.
	B	Beta		
(Constante)	-0.640		-3.760	0.000*
ACA	0.063	0.304	9.517	0.000*
Age	-0.004	-0.317	-9.419	0.000*
KmF	0.037	0.318	9.672	0.000*
Sex	-0.042	-0.122	-3.848	0.000*
Ele F Apex	-0.032	-0.217	-4.658	0.000*
Ele F Thin	0.015	0.154	3.446	0.001*

R^2 (%)= 31.3%   Model F = 57.542

* Statistically significant ($p<0.05$)

Table S7.- Multivariant regression Model 2 for the RMS total of the posterior cornea.

Dependent variables predicting RMS total (Posterior cornea)	Unstandardized coefficients	Standardized coefficients	t	Sig.
	B	Beta		
(Constante)	-0.519		-4.577	0.000*
PCA	0.420	0.418	16.103	0.000*
QB	0.263	0.337	7.350	0.000*
KmB	-0.210	-0.309	-11.982	0.000*
Ele B Thin	0.014	0.464	11.615	0.000*
Ele B Apex	-0.028	-0.560	-10.283	0.000*
Age	-0.002	-0.144	-5.094	0.000*
Sex	-0.023	-0.065	-2.809	0.005*
RA	-0.011	-0.061	-2.549	0.011*

R^2 (%)= 63.6%   Model F = 147.576

* Statistically significant ($p<0.05$)

Table S8.- Multivariant regression Model 3 for the RMS total of the total cornea.

Dependent variables predicting RMS total (Total cornea)	Unstandardized coefficients	Standardized coefficients	t	Sig.
	B	Beta		
(Constante)	0.350		0.332	0.740
ACA	0.555	0.582	19.462	0.000*
Age	0.011	0.208	6.776	0.000*
Ele F Apex	-0.203	-0.305	-6.880	0.000*
RA	-0.179	-0.213	-7.281	0.000*
WTW	-0.158	-0.085	-3.209	0.001*
Ele F Thin	-0.063	-0.144	-4.332	0.000*
QF	-0.885	-0.149	-3.875	0.000*
QB	-0.438	-0.123	-4.057	0.000*
KmF	0.093	0.174	3.442	0.001*
KmB	0.306	0.098	1.996	0.046*

R^2 (%)= 63.7% Model F = 132.181

* Statistically significant ($p<0.05$)

Table S9.- Multivariant regression Model 3 for the RMS LOA of the total cornea.

Dependent variables predicting RMS LOA (Total cornea)	Unstandardized coefficients	Standardized coefficients	t	Sig.
	B	Beta		
(Constante)	0.038		0.037	0.971
ACA	0.562	0.592	19.887	0.000*
Age	0.012	0.222	7.700	0.000*
Ele F Apex	-0.201	-0.304	-6.947	0.000*
RA	-0.185	-0.220	-7.549	0.000*
WTW	-0.136	-0.073	-2.803	0.005*
QF	-1.045	-0.176	-4.642	0.000*
Ele F Thin	-0.061	-0.140	-4.233	0.000*
QB	-0.308	-0.087	-3.219	0.001*
KmF	0.046	0.087	3.218	0.001*

R^2 (%)= 63.9% **Model F = 134.182**

* Statistically significant ($p<0.05$)
